# Docosahexaenoic Acid Induces Expression of Heme Oxygenase-1 and NAD(P)H:quinone Oxidoreductase through Activation of Nrf2 in Human Mammary Epithelial Cells

**DOI:** 10.3390/molecules22060969

**Published:** 2017-06-10

**Authors:** Hye-Yoon Bang, Sin-Aye Park, Soma Saeidi, Hye-Kyung Na, Young-Joon Surh

**Affiliations:** 1 Tumor Microenvironment Global Core Research Center and Research Institute of Pharmaceutical Sciences, Seoul National University, Seoul 08826 Korea; bangdeng1126@naver.com (H.-Y.B.); qorwh7@snu.ac.kr (S.-A.P.); Saeidi@snu.ac.kr (S.S.); 2 Cancer Research Institute, Seoul National University, Seoul 03080, Korea; 3 Department of Molecular Medicine and Biopharmaceutical Sciences, College of Pharmacy, Seoul National University, Seoul 08826, Korea; 4 Department of Food and Nutrition, College of Human Ecology, Sungshin Women’s University, Seoul 01133, Korea; nhkdec28@gmail.com

**Keywords:** docosahexaenoic acid, ω-3 polyunsaturated fatty acids, heme oxygenase-1, NAD(P)H:quinone oxidoreductase, nrf2

## Abstract

Docosahexaenoic acid (DHA), an ω-3 fatty acid abundant in fish oils, has diverse health beneficial effects, such as anti-oxidative, anti-inflammatory, neuroprotective, and chemopreventive activities. In this study, we found that DHA induced expression of two representative antioxidant/cytoprotective enzymes, heme oxygenase-1 (HO-1) and NAD(P)H:quinone oxidoreductase (NQO1), in human mammary epithealial (MCF-10A) cells. DHA-induced upregulation of these enzymes was accompanied by enhanced translocation of the redox-sensitive transcription factor Nrf2 into the nucleus and its binding to antioxidant response element. Nrf2 gene silencing by siRNA abolished the DHA-induced expression of HO-1 and NQO1 proteins. When MCF-10A cells were transfected with mutant constructs in which the cysteine 151 or 288 residue of Keap1 was replaced by serine, DHA-induced expression of HO-1 and NQO1 was markedly reduced. Moreover, DHA activated protein kinase C (PKC)δ and induced Nrf2 phosphorylation. DHA-induced phosphorylation of Nrf2 was abrogated by the pharmacological PKCδ inhibitor rottlerin or siRNA knockdown of its gene expression. The antioxidants *N*-acetyl-l-cysteine and Trolox attenuated DHA-induced activation of PKCδ, phosphorylation of Nrf2, and and its target protein expression. In conclusion, DHA activates Nrf2, possibly through modification of critical Keap1 cysteine 288 residue and PKCδ-mediated phosphorylation of Nrf2, leading to upregulation of HO-1 and NQO1 expression.

## 1. Introduction

The two representative omega-3 polyunsaturated fatty acids (n-3 PUFAs), eicosapentaenoic acid (EPA) and docosahexaenoic acid (DHA), abundant in some fish oils, possess anti-oxidative [1,2], anti-inflammatory [3], neuroprotective [4], and chemopreventive [5] effects. While n-6 PUFAs are associated with the increased probability of a number of diseases [6,7,8], n-3 PUFAs have been reported to have protective effects against initiation and promotion of experimentally induced carcinogenesis [9,10]. A double-blind randomized study with healthy volunteers showed that dietary supplementation with n-3 PUFAs protected against UV-induced DNA damage in peripheral blood lymphocytes [2]. Furthermore, n-3 PUFA supplementation during pregnancy improved infant development of the brain and neuronal retina [11]. Several clinical trials suggest that n-3 PUFA supplementation during cancer chemotherapy improves patient outcomes [12]. The overall survival of breast cancer patients with high incorporation of DHA almost doubled compared to patients with low incorporation of DHA [13]. Supplementation with n-3 PUFA also increased therapeutic response in patients with advanced non-small cell lung cancer [14]. Although n-3 PUFAs are very susceptible to free radical attack or oxidation [15], paradoxically their protective effects against oxidative stress and injury have also been reported [2,3]. DHA inhibits lipopolysaccharide (LPS)-induced expression of tumor necrosis factor alpha and interleukin-1β and also has an inhibitory effect on LPS-induced IκB degradation and subsequent nuclear translocation of p65, a functionally active subunit of NF-κB [16]. The mixture of EPA and DHA suppresses the pro-inflammatory response in LPS-stimulated mouse microglia by activating deacetylase sirtuin1 [17]. In a mouse model of cigarette smoke (CS)-induced endothelial dysfunction, n-3 PUFA-enriched diet decreased CS-induced production of an oxidative stress marker, 8-isoprostane and significantly reduced the mRNA level of cytochrome P4501A1 [18]. Additionally, n-3 PUFA-enriched diet protected mice from Concanavalin A-induced liver injury and suppressed the expression of pro-inflammatory cytokines [19]. However, the molecular mechanisms underlying anti-oxidative and anti-inflammatory effects of n-3 PUFAs in relation to their chemopreventive potential remain largely unresolved.

Nuclear factor E2-related factor (Nrf2) is a redox-sensitive transcription factor responsible for regulating the expression of genes encoding many anti-oxidant enzymes and cytoprotective proteins, such as heme oxygenase-1 (HO-1), NAD(P)H:quinone oxidoreductase-1 (NQO1), glutamate-cysteine ligase, glutathione *S*-transferases, superoxide dismutase, glutathione peroxidase, uridine diphosphate glucuronosyltransferase, etc. [20,21]. The antioxidant response element (ARE), a *cis*-acting DNA sequence, is located in the promoter region of genes encoding these proteins [22]. Under normal physiological conditions, Nrf2 is sequestered in the cytoplasm by its repressor, Kelch-like ECH associated protein 1 (Keap1). As an adaptive response to oxidative stress or electrophilic insults, Nrf2 is dissociated from Keap1 and translocates to nucleus, regulating the transcription of cytoprotective genes by binding to ARE [23]. Many electrophilic lipid mediators and their oxidized products, such as 15-deoxy-Δ^12,14^-prostagandin J_2_ [24], 4-hydroxynonenal [25], and 15-J_2_-isoprostane [26], have been reported to activate Nrf2 and induce expression of cytoprotective enzymes.

The oxidative modification of certain cysteine thiol groups present in Keap1 plays a key role in liberating Nrf2. Some specific cysteine residues of Keap1 have been proposed to be covalently modified by electrophilic agents or chemopreventive phytochemicals [27,28]. Furthermore, several protein kinases have been found to phosphorylate Nrf2 at Ser 40, thereby stimulating ARE-driven transcription [29]. Among them, protein kinase C (PKC) family members have been reported to mediate nuclear localization of Nrf2 in response to chemopreventive agents [30,31]. The present study was intended to examine the effects of EPA and DHA on Nrf2 activation, in the context of their antioxidant, anti-inflammatory and chemopreventive activities.

## 2. Results

### 2.1. DHA Upregulates Expression of HO-1 and NQO1

**Figure 1:**
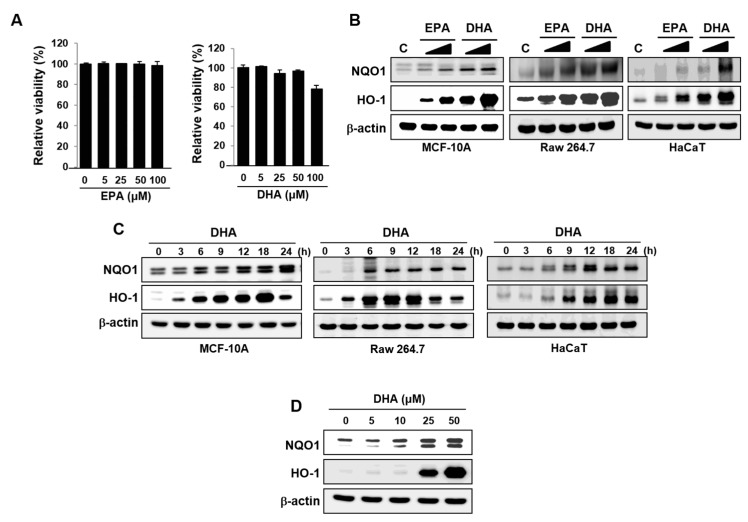
Effects of DHA on the expression of HO-1 and NQO1. (**A**) MCF-10A cells were treated with EPA or DHA for 48 h with indicated concentrations. Cell viability was determined in triplicate by the MTT assay. Columns, means (n = 3); bars, standard deviation (SD). (**B**) MCF-10A, Raw 264.7, or HaCaT cells were incubated with EPA or DHA (25 or 50 μM) for 18 h. Whole cell lysates were subjected to Western blot analysis. (**C**) MCF-10A, Raw 264.7, or HaCaT cells exposed to DHA (25 μM) were harvested at the indicated intervals, and the protein expression was assessed by Western blot analysis. (**D**) MCF-10A cells were treated with indicated concentrations of DHA for 18 h, and the expression levels of HO-1 and NQO1 were measured by Western blot analysis.

HO-1 and NQO1 are prototypic antioxidant enzymes that are known to play a key role in cellular cytoprotection against oxidative stress [32]. In comparing the effects of EPA and DHA on the expression of HO-1 and NQO1, we first verified that both n-3 PUFAs elicited no substantial cytotoxicity at a concentration up to 100 μM (Figure 1A). When MCF-10A cells were incubated with 25 μM DHA, there was a progressive increase in the protein expression of HO-1 and NQO1.

EPA also induced expression of both enzymes, but the effects were much weaker than those achieved with DHA (Figure 1B). These results were confirmed in other normal cell lines including mouse macrophage Raw 264.7 and human adult keratinocytes HaCaT cells (Figure 1B). DHA induced the expression of HO-1 and NQO1 in all these three cell lines in a time-dependent manner (Figure 1C). In addition, treatment of MCF-10A cells with DHA induced expression of HO-1 and NQO1 in a concentration-dependent manner (Figure 1D). As DHA exerted stronger effects than EPA in expression of antioxidant enzymes, the subsequent mechanistic studies were conducted with the former n-3 PUFA.

### 2.2. DHA Induces the Nuclear Translocation and Transcriptional Activity of Nrf2 through ARE Binding

**Figure 2:**
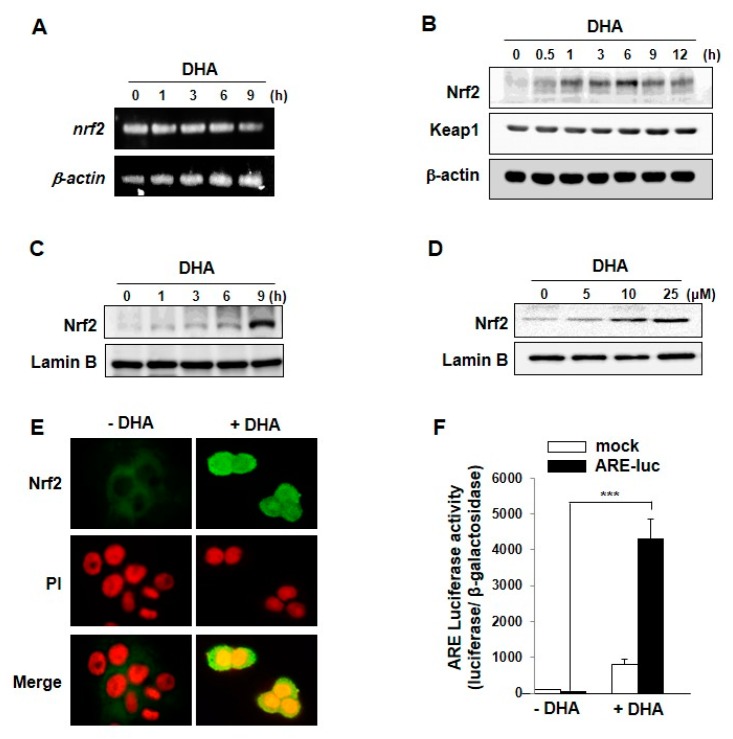
DHA-induced expression, nuclear translocation and transcriptional activity of Nrf2. (**A**) Total RNA was isolated from cells treated with or without DHA for indicated duration and analyzed by RT-PCR for detecting the level of Nrf2 mRNA; (**B**) MCF-10Acells were exposed to DHA (25 μM) were harvested at the indicated intervals, and the protein levels were assessed by Western blot analysis. (**C**) Nuclear extracts from MCF-10A cells were prepared at the indicated intervals after treatment with DHA (25 μM). (**D**) MCF-10A cells were treated with indicated concentrations of DHA for 9 h and the nuclear translocation of Nrf2 was assessed by Western blot analysis. (**E**) MCF-10A cells were incubated with DHA (25 μM) for 9 h and nuclear localization of Nrf2 was determined by immunocytochemical analysis. (**F**) MCF-10A cells were treated with DHA (25 μM) for 9 h after transfection with either an ARE luciferase construct or a control vector and analyzed for the Nrf2 transcriptional activity as described in Materials and Methods. Columns, means (n = 3); bars, SD. ***, *p* < 0.001.

As Nrf2 is involved in transcriptional regulation of many cytoprotective proteins including HO-1 and NQO-1, we determined whether DHA could stimulate nuclear translocation of this transcription factor and its transcriptional activity. DHA did not influence the Nrf2 mRNA level (Figure 2A), while its treatment enhanced the Nrf2 protein expression as assessed by immunoblot analysis (Figure 2B). In addition, DHA increased the nuclear accumulation of Nrf2 in time- (Figure 2C) and concentration-dependent (Figure 2D) manners in MCF-10A cells. DHA-induced nuclear translocation of Nrf2 was further verified by immunocytochemical analysis (Figure 2E). We also examined the effect of DHA on ARE luciferase activity. The induction of Nrf2 transcriptional activity as assessed by the luciferase reporter gene assay was markedly enhanced after treatment with DHA for 9 h (Figure 2F).

### 2.3. DHA-Induced Expression of HO-1 and NQO1 Is Mediated by Nrf2

**Figure 3:**
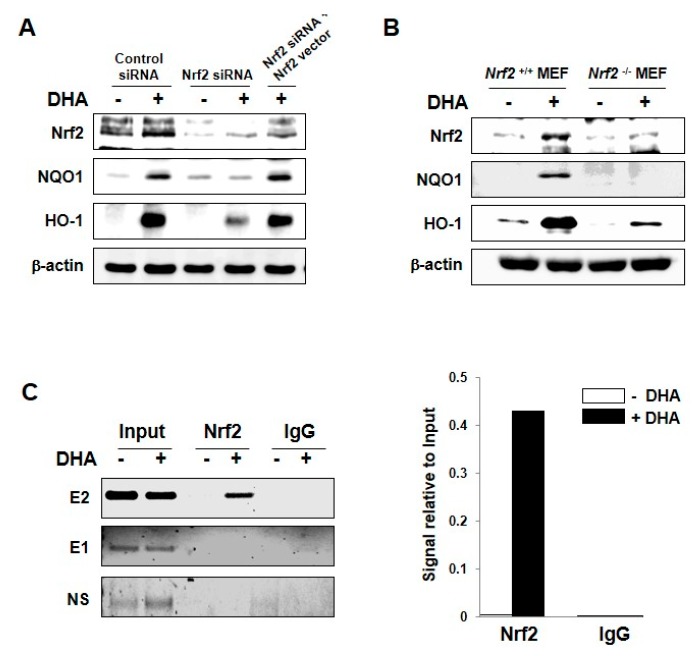
Role of Nrf2 in DHA-induced expression of HO-1 and NQO1. (**A**) MCF-10A cells were transfected with siRNA control, siRNA Nrf2, or siRNA Nrf2 plus Nrf2 full-sequence vector for 12 h and exposed to DHA (25 μM) for another 9 h. Whole cell lysates were subjected to Western blot analysis. (**B**) Nrf2-WT or Nrf2-null MEF cells were incubated with 25 μM of DHA for 12 h, and the expression of Nrf2, HO-1, and NQO1 was measured by Western blot analysis. (**C**) MCF-10A cells were treated with DHA (25 μM) for 9 h and harvested to determine the ARE binding activity by the ChIP assay. Chromatin immunoprecipitated DNA was analyzed by RT-PCR with primers for distal E2 (−9.0 kb region) and E1 (−4.0 kb region) AREs as well as non-specific region (after −9.0 kb) of the HO-1 promoter.

To determine whether upregulation of target gene transcription is mediated by Nrf2, we examined the expression of HO-1 and NQO1 after siRNA-mediated knockdown of Nrf2. Silencing of Nrf2 by use of small interfering RNA abolished the expression of these enzymes induced by DHA, which was restored by transfection of full length Nrf2 vector (Figure 3A). In addition, when embryonic fibroblasts (MEFs) prepared from Nrf2 null mice were incubated with DHA, it failed to induce expression of HO-1 and NQO1 while Nrf2^+/+^ MEFs were responsive to DHA (Figure 3B). We also performed the chromatin immunoprecipitation (ChIP) assay to ensure that Nrf2 directly binds to the promoter of the HO-1 gene. In Nrf2-immunoprecipitated samples, a clear band was observed when the cells were exposed to DHA, corroborating the direct binding of Nrf2 to only the distal E2 (−9.0 kb), an ARE rich region of HO-1 promoter (Figure 3C). However, Nrf2 failed to bind the E1 region (−4.0 kb) and a non-specific region (after −9.0 kb) of the HO-1 promoter upon DHA treatment.

### 2.4. Cysteine Residues of Keap1 May Be Putative Targets of DHA for Its Induction of Nrf2-Driven Expression of HO-1 and NQO1

**Figure 4:**
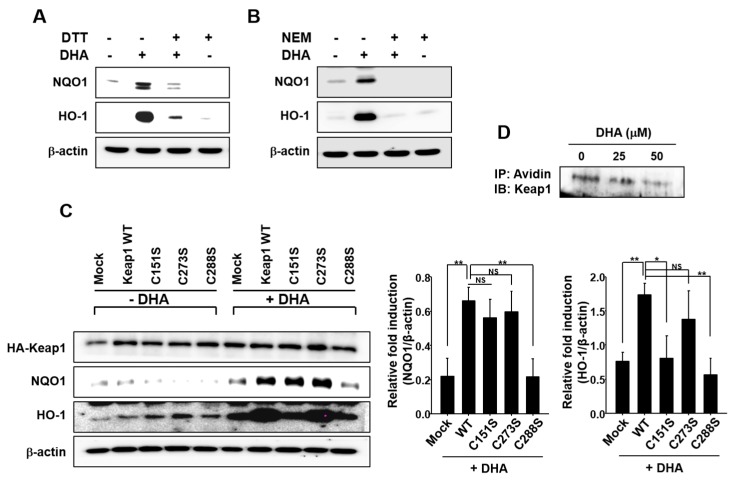
Possible involvement of Keap1 cysteine thiol modification in DHA-induced HO-1 and NQO1 expression. (**A**) MCF-10A cells were pretreated with or without a thiol reducing agent DTT (500 μM) for 1 h, followed by 18-h incubation with 25 μM DHA. (**B**) Cells were pretreated with another thiol reducing agent NEM (25 μM) for 1 h, followed by 18-h incubation with 25 μM DHA. (**C**) MCF-10A cells were transfected with HA-mock, HA-Keap1 WT, Keap1-C151S, Keap1-C273S, or Keap1-C288S expressing vector for 24 h, and incubated with DHA (25 μM) for another 9 h to determine the expression of HO-1 and NQO1. HA-Keap1 was used to ensure the equal expression of mutant vectors. Each blot is a representative of three different experiments. Columns, means (n = 3); bars, SD. *, *p* < 0.05 or **, *p* < 0.01. NS. non-significant. (**D**) MCF-10A cells were incubated with DHA (25 or 50 μM) for 6 h and then treated with RIPA lysis buffer. Cell lysates were incubated with BPM for 30 min, and then immunoprecipitated with avidin-agarose. The precipitated proteins were subjected to Western blot analysis with an anti-Keap1 antibody.

Several studies have shown that some phytochemicals and electrophilic compounds directly interact with Keap1 at its specific thiol group(s), facilitating the release of Nrf2 for nuclear translocation [33]. As an initial step towards determining whether such cysteines thiol modification is involved in DHA-induced activation of Nrf2 in MCF-10A cells, the cells were incubated with DHA with or without the thiol reducing agent dithiothreitol (DTT). DTT treatment abrogated the DHA-induced HO-1 and NQO1 expression (Figure 4A). The thiol alkylating agent *N*-ethylmaleimide (NEM) also significantly inhibited DHA-induced expression of HO-1 and NQO1 (Figure 4B).

Human Keap1 has 27 cysteine residues, some of which are known to be responsible for regulation of Nrf2 [27,34]. Among these, cysteine 151, 273, and 288 are considered to be redox sensors. To determine which of these cysteine residue(s) play(s) a role in Nrf2 activation by DHA, MCF-10A cells were transiently transfected with HA-Keap1 WT or each of the mutant vectors in which aforementioned Keap1 cysteine residues were replaced by serine. As illustrated in Figure 4C, a single amino acid change from Cys to Ser at position 151 or 288 abolished DHA-induced HO-1 expression. However, the DHA-induced NQO1 expression was reduced by the transfection of Keap1-C288S plasmid, not by Keap1-C151S. In addition, treatment of MCF-10A cells with DHA reduced the level of Keap1 modified by biotin-PEAC_5_-maleimide (BPM) in a concentration-dependent manner, corroborating the covalent modification of Keap1 in cells treated with DHA (Figure 4D).

### 2.5. DHA-Derived Intracellular Reactive Oxygen Species (ROS) Accumulation Induces Phosphorylation of Nrf2 via the PKCδ Signaling

**Figure 5:**
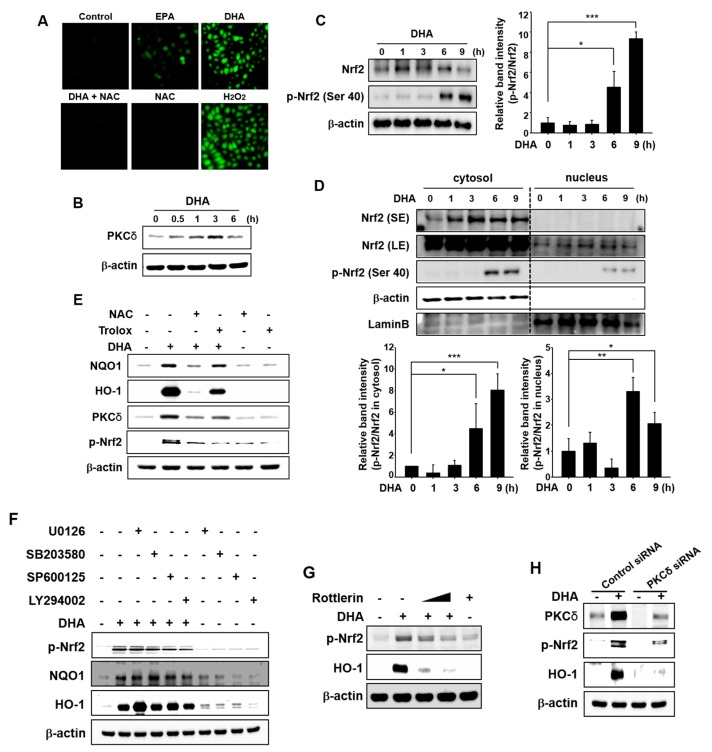
Role of ROS in DHA-induced Nrf2 activation mediated by PKCδ. (**A**) MCF-10A cells were treated with each indicated drug (EPA and DHA; 25 μM, NAC; 5 mM, H_2_O_2_; 100 μM) or in proper combination for 3 h and then examined for the intracellular accumulation of ROS under the confocal microscope after DCF-DA fluorescence staining. (**B**) Cells were treated with DHA (25 μM) for the indicated time periods, and the effect of DHA on PKCδ activation was determined by Western blot analysis. (**C**) MCF-10A cells treated with DHA (25 μM) were harvested at the indicated time intervals. The effect of DHA on the phosphorylation of Ser 40 at Nrf2 was assessed by Western blot analysis. Each blot is a representative of three different experiments. Columns, means (n = 3); bars, SD. * *p* < 0.05 or *** *p* < 0.001. (**D**) Nuclear and cytosol extracts were prepared at the indicated intervals after incubation with DHA (25 μM). Each blot is a representative of three different experiments. Columns, means (n = 3); bars, SD. * *p* < 0.05 or ** *p* < 0.01 or *** *p* < 0.001. SE; short exposure, LE; long exposure. (**E**) Cells were pretreated with NAC (5 mM) or Trolox (100 μM) for 1 h, then incubated with DHA (25 μM) for another 3 h (for PKCδ), 6 h (for p-Nrf2) or 9 h (for HO-1 and NQO1). (**F**) Cells were pretreated with U0126, SB203580, SP600125, or LY294002 (25 μM) for 1 h, followed by incubation with DHA (25 μM) for additional 6 h (for p-Nrf2) or 18 h (for HO-1 and NQO1). (**G**) Cells were pretreated with rottlerin (1 or 10 μM) for 1 h, and then incubated with DHA (25 μM) for additional 3 h. (**H**) MCF-10A cells were treated with DHA (25 μM) for 6 h after transfection with siRNA control or siRNA PKCδ for 24 h.

Besides thiol modification of Keap1, phosphorylation of Nrf2 has also been considered to be essential for activation of this transcription factor. DHA is anticipated to act as a pro-oxidant by producing moderate amounts of ROS through auto-oxidation [35], which may lead to activation of Nrf2. Therefore, we examined DHA-induced ROS production by 2′,7′-dichlorofluorescin diacetate (DCF-DA) staining. Incubation with EPA or DHA resulted in a modest increase in the intracellular accumulation of ROS as compared to control. DHA-induced ROS production was much higher than that achieved with EPA in MCF-10A cells (Figure 5A). Pretreatment with *N*-acetyl-l-cysteine (NAC) abolished DHA-induced ROS generation. In this experiment, H_2_O_2_ was used as a positive control.

Activation of signal transducing kinases including mitogen-activated protein kinases (MAPKs), phosphoinositide 3-kinase (PI3K)/Akt, and PKC has been reported to induce nuclear localization of Nrf2 and subsequent expression of HO-1 and NQO1. Thus, PKC phosphorylates Nrf2, thereby inducing nuclear translocation of Nrf2 and expression of antioxidant enzymes in response to oxidative stress [36]. PKCδ phosphorylates Nrf2 at Ser 40, which facilitates its release from Keap1 [29]. To investigate the upstream signaling events responsible for the activation of Nrf2 and induction of HO-1 expression, we first examined the effect of DHA on the induction of PKCδ. DHA induced PKCδ expression in MCF-10A cells (Figure 5B). We found that phosphorylation of Nrf2 (Ser 40) was increased following treatment with DHA (Figure 5C). Nuclear translocation of phosphorylated Nrf2 (p-Nrf2) was also increased by DHA treatment in a time-dependent manner (Figure 5D). To verify that the DHA-induced intracellular accumulation of ROS activates PKCδ-Nrf2 signaling, MCF-10A cells were incubated with NAC or Trolox. As illustrated in Figure 5E, NAC strongly reduced the expression levels of PKCδ, HO-1, NQO1, and p-Nrf2 in DHA-treated cells. Likewise, Trolox decreased the expression level of p-Nrf2, PKCδ, HO-1, and NQO1 in DHA-treated cells, but to a lesser extent than that achieved with NAC (Figure 5E). Pretreatment with U0126 (inhibitor of ERK), SB203580 (inhibitor of p38 MAPK), SP600125 (inhibitor of JNK), and LY2940002 (inhibitor of PI3K/Akt) failed to abrogate DHA-induced phosphorylation of Nrf2 and expression of HO-1 and NQO1 (Figure 5F). These findings suggest that MAPKs and Akt are not involved in DHA-induced activation of Nrf2. As expression of PKCδ was induced by DHA treatment, we examined whether this kinase is involved in phosphorylation of Nrf2. For this purpose, we used rottlerin, a pharmacologic inhibitor of PKCδ. As shown in Figure 5G, co-treatment with rottlerin abrogated DHA-induced phosphorylation of Nrf2 and expression of HO-1. Likewise, DHA-induced expression of p-Nrf2 and HO-1 was abolished in PKCδ-knockdown cells (Figure 5H).

## 3. Discussion

Oxidative stress, the outcome of an imbalance between the generation of ROS and the cellular antioxidant abilities, contributes to various chronic diseases including cancer [37]. It has been well recognized that the induction of antioxidant enzymes confers a first-line defense against deleterious effects of oxidative stress and other toxicants [38,39]. HO-1 and NQO1 are representative antioxidant/cytoprotective enzymes that are induced in response to a wide range of stimuli that cause oxidative stress and pathological conditions [24,40,41]. Many edible natural compounds have been shown to upregulate the expression of these cytoprotective enzymes, which accounts for their anti-oxidative, anti-inflammatory, anti-carcinogenic and chemopreventive effects [42,43]. DHA is a representative n-3 PUFA abundant in fish oil that has a wide array of health beneficial effects. However, the excessive accumulation of n-3 PUFAs including DHA increases the production of free radicals, enhances oxidative stress, and causes pathologic changes in some specific organs [44,45,46]. These findings suggest that even n-3 PUFAs should be carefully considered with regards to their dosage, duration, and target organ.

In the present study, we found that DHA induces activation of Nrf2-ARE signaling, and subsequently expression of HO-1 and NQO1. Under basal conditions, Keap1 impedes the nuclear localization of Nrf2 by sequestering it in the cytoplasm. Oxidative stress or some chemopreventive agents can cause the dissociation of Nrf2 from Keap1 through oxidation or covalent modification of specific cysteine residue(s) of Keap1 which facilitates dissociation of Nrf2 from Keap1 and nuclear translocation [47,48,49]. Our results demonstrate that DHA treatment to MCF-10A cells enhanced the nuclear translocation of Nrf2 and its ARE binding, thereby stimulating transcriptional activity of Nrf2.

Under physiological conditions, DHA can be oxidized enzymatically or non-enzymatically. Since DHA is the longest and most unsaturated fatty acid among PUFAs, it is more prone to oxidation than EPA or arachidonic acid [50]. In the enzymatic oxidation pathway, cyclooxygenase-2 catalyzes conversion of DHA to 17-hydroxy-DHA, which is released from the endothelium and converted to electrophilic 17-oxo-DHA by dehydrogenase activity. Non-enzymatic peroxidation of DHA produces the highly electrophilic species, such as A_4_/J_4_-neuroprostances and *trans*-4-hydroxy-2-hexenal, which possess an α,β-unsaturated carbonyl moiety capable of activating Nrf2 signaling [51,52,53,54]. In our present study, mutations of Cys288 in Keap1 abolished DHA-induced expression of both HO-1 and NQO1 expression, whilst Cys151 mutation affected expression of only HO-1. As DHA lacks the α,β-unsaturated moiety, this finding suggests that electrophilic species derived from DHA may act as Michael acceptors that can directly bind to the nucleophilic cysteine thiols located in Keap1, thereby facilitating Nrf2 activation. The identification of reactive metabolites/species involved in DHA-induced Nrf2 activation needs further investigation.

Besides direct interaction with Keap1 cysteines that are considered sensors of oxidative and electrophilic stresses, DHA may activate Nrf2 via other mechanisms. ROS-mediated activation of several protein kinases including MAPKs or PKCδ results in phosphorylation of Nrf2, which facilitates dissociation of Nrf2 from Keap1 [29,55]. Several previous studies suggest that phosphorylation of Nrf2 at Ser 40 increases the response of Nrf2 to chemical inducers and nuclear accumulation of Nrf2 [56,57]. In our present study, DHA failed to increase the expression of MAPKs, such as ERK1/2JNK, and p38 kinase (data not shown). Moreover, DHA promotes phosphorylation of Akt (data not shown), but inhibition of its kinase activity does not block the expression of HO-1 and NQO1. However, DHA upregulated the expression of PKCδ, and inhibition of its activity using rottlerin or siRNA blocked DHA-induced p-Nrf2 and HO-1 expression. The attenuation of DHA-induced activation of PKCδ and Nrf2, and subsequent expression of HO-1 upon pretreatment of cells with NAC or Trolox suggest that DHA-induced ROS production may also play a role in turning on the Nrf2 signaling. NAC is a precursor of glutathione that can react directly with ROS, but also can act as the thiol reducing agents like DTT, which converts cystine to cysteine while Trolox is a water-soluble derivative of vitamin E and non-thiol reducing antioxidant [58]. Compared with NAC, Trolox only partially inhibited DHA-induced HO-1 and NQO1 expression. These findings suggest that DHA-induced upregulation of HO-1 and NQO1 may be attributable to both covalent modification and oxidation of cysteine present in Keap1.

## 4. Materials and Methods

### 4.1. Materials

EPA and DHA (each purity > 98%) were purchased from Cayman Chemical Co. (Ann Arbor, MI, USA). EPA and DHA were supplied as a solution in ethanol. Ethanol was evaporated under a gentle stream of nitrogen and the sterile dimethylsulfoxide (DMSO) was added to prepare 100 mM stock solution. Further dilutions of the stock solution were made prior to performing experiments. Trizol^®^ was a product of GIBCO BRL (Grand Island, NY, USA). (3-(4,5-dimethylthiazol-2-yl)-2,5-diphenyltetrazoliumbromide) (MTT), DTT, NAC, rottlerin, LY294002, U0126, SB203580, and SP600125 were purchased from Sigma Chemical Co. (St. Louis, MO, USA). Rabbit polyclonal HO-1 antibody was the product of Stressgen (Ann Arbor, MI, USA). Primary antibodies for Nrf2, Keap1, normal mouse IgG, PKCδ siRNA (sc-36253), and protein G plus-agarose were supplied by Santa Cruz Biotechnology (Santa Cruz, CA, USA). Anti-HA, anti-PKCδ, and Horseradish peroxidase (HRP)-linked anti-biotin antibodies were obtained from Cell Signaling Technology (Beverly, MA, USA). Anti-rabbit and anti-mouse HRP-secondary antibodies were provided by Zymed Laboratories Inc. (San Francisco, CA, USA). Antibodies against NQO1 were obtained from Abcam Technology (Cambridge, UK) or Santa Cruz Biotechnology (Santa Cruz, CA, USA). The phospho-Nrf2 (p-Nrf2) were from Abcam Technology (Cambridge, UK). The human specific Nrf2-siRNA (sense 5′-AAGAGUAUGAGCUGGAAAAACTT-3′; antisense 5′-GUUUUUCCAGCUCAUACU-CUUTT-3′); stealth^™^ RNAi negative control duplexes, and 2′,7′-dichlorofluorescein diacetate (DCF-DA) were provided by Invitrogen (Carlsbad, CA, USA). Hank’s balanced salt solution (HBSS) was purchased from Meditech, Inc. (Herndon, VA, USA). BPM was obtained from Dojindo (Kumamoto, Japan).

### 4.2. Cell Culture

Human breast epithelial MCF-10A cells, mouse macrophage Raw 264.7 cells, and human adult keratinocyte HaCaT cells were obtained from the American Type Culture Collection. All cells have been passaged directly from the original low-passage stocks and were used before passage 30. The cells were also tested within the last three months for correct morphology under microscope and tested to detect any mycoplasma contamination using an e-Myco^™^ plus mycoplasma PCR detection kit (iNtRON Biotechnology, Seongnam, Korea). Cells were maintained at 37 °C in a humidified atmosphere of 5% CO_2_ and 95% air.

### 4.3. Preparation and Culturing of MEF

The Nrf2 wild type (*Nrf2^+/+^*) and Nrf2-null (*Nrf2**^−/−^***) mice were provided by Dr. Jeffery Johnson (University of Wisconsin-Madison, Madison, WI, USA). After in-house breeding, the *Nrf2**^−/−^***, *Nrf2^+/**−**^* and wild-type mice were maintained in the animal quarters in accordance with Seoul National University guidelines for animal care and were housed in a 12-h light/12-h dark cycle. They were fed standard rodent chow and given water ad libitum. Male and female *Nrf2^+/**−**^* mice were paired and the pregnancies were monitored. Embryos were obtained at the day 13 after pairing under aseptic conditions. The heads of the embryos were used to confirm the *nrf2* genotype by reverse transcriptase-PCR (RT-PCR), and the embryo bodies were minced into small pieces and cultured in high glucose DMEM supplemented with 10% fetal bovine serum and kept at 37 °C with 5% CO_2_.

### 4.4. Preparation of Nuclear Extracts

The cells were washed with ice-cold phosphate-buffered saline (PBS), scraped in PBS and centrifuged at 13,000 *g* for 15 min at 4 °C. Pellets were suspended in hypotonic buffer A (10 mM HEPES (pH 7.8), 1.5 mM MgCl_2_, 10 mM KCl, 0.5 mM DTT, and 0.2 mM phenylmethylsulfonyl fluoride (PMSF)) for 15 min on ice, and 10% Nonidet P-40 solution was added for 5 min. The mixture was centrifuged at 12,000 *g* for 10 min. The pellets were washed with hypotonic buffer and resuspended in hypertonic buffer C (20 mM (HEPES (pH 7.8), 20% glycerol, 420 mM NaCl, 1.5 mM MgCl_2_, 0.2 mM ethylenediaminetetraacetic acid (EDTA), 0.5 mM DTT, and 0.2 mM PMSF) for 30 min on ice and centrifuged at 12,000 *g* for 10 min. The supernatant containing nuclear proteins was collected and stored at −80 °C.

### 4.5. Western Blot Analysis

The cells were washed with PBS and incubated with cell lysis buffer (50 mM Tris-HCl (pH 7.4), 150 mM NaCl, 25 mM NaF, 20 mM ethylenediaminetetraacetic acid, 1 mM DTT, 1 mM Na_3_VO_4_, 0.5% Triton X-100 and protease inhibitor cocktail tablets) for 1 h on ice, followed by centrifugation at 13,000 rpm for 15 min. The protein concentration of the supernatant was measured by using the bicinchoninic acid reagents. The protein samples were solubilized with sodium dodecyl sulfate (SDS)-polyacrylamide gel electrophoresis sample loading buffer and boiled for 5 min. Proteins were electrophoresed on 8% or 10% SDS-polyacrylamide gel and transferred to polyvinylidene difluoride membranes. The blots were then blocked with 5% fat free dry milk-TBST (Tris-buffered saline containing 0.1% Tween-20) buffer for 1 h at room temperature and incubated with primary antibodies (dilution in 1:1000) in 3% fat-free dry milk-TBST. Following three washes with TBST, the blots were incubated with horseradish peroxidase-conjugated secondary antibody (dilution in 1:4000) in 3% fat-free dry milk-TBST for 1 h at room temperature. The blots were rinsed again three times with TBST, and the transferred proteins were incubated with ECL substrate solution (Amersham Pharmacia Biotech, Piscataway, NJ, USA) for 1 min according to the manufacturer’s instruction and visualized with LAS 4000 (Fuji film, Tokyo, Japan) [59].

### 4.6. RT-PCR

Total RNA was isolated from cells using Trizol^®^. One microgram of total RNA was used for the complementary DNA synthesis using random primers. RT-PCR was performed following standard procedures. For detection of *Nrf2* mRNA, 20 cycles of 94 °C for 30 s, 57 °C for 30 s, and 68 °C for 1 min were conducted; for quantitation of *actin* mRNA, 20 cycles of 94 °C for 30 s, 59 °C for 35 s, and 72 °C for 30 s were conducted. These PCR cycles were followed by a final extension for 7 min at 72 °C. The primers used for each RT-PCR reactions are as follows: Nrf2, 5′-GCG ACG GAA AGA GTA TGA GC-3′ and 5′-GTT GGC AGA TCC ACT GGT TT-3′; actin, 5′-AGA GCA TAG CCC TCG TAG AT-3′ and 5′-CCC AGA GCA AGA GAG GTA TC-3′.

### 4.7. ARE Luciferase Assay

MCF-10A cells were plated in a 6-well plate and co-transfected with 1.5 μg of the luciferase reporter gene fusion construct (pTi-luciferase), wild-type ARE, and 0.5 μg of pCMV-β-galactosidase control vector with WelFect-M^™^ Gold transfection reagent (WelGENE Inc., Gyeongsan, Korea) according to the manufacturer’s instructions. After 24-h transfection, the cells were treated with DHA for additional 9 h, and cell lysis was carried out with the 1× reporter lysis buffer (Promega, Madison, WI, USA). After mixing the cell extract with a luciferase substrate (Promega, Madison, WI, USA), the luciferase activity was measured by the luminometer (AntoLumat LB 953, EG&G Berthold, Bad Widbad, Germany). The β-galactosidase assay was done according to the supplier’s instructions (Promega, Madison, WI, USA) for normalizing the luciferase activity.

### 4.8. Immunocytochemistry

MCF-10A cells were plated on the chamber slide and treated with DHA or vehicle alone. After fixation with 10% neutral-buffered formalin solution for 30 min at room temperature, samples were incubated with blocking agents (0.1% Tween-20 in PBS containing 5% bovine serum albumin), washed with PBS and then incubated with a diluted (1:100) primary antibody overnight at 4 °C. After washing with PBS, samples were incubated with a diluted (1:1000) FITC-goat anti-rabbit IgG secondary antibody for 1 h and examined under a confocal microscope (Leica, Wetzlar, Germany).

### 4.9. ChIP Assay

DNA and proteins of the cells were cross-linked in 37% formaldehyde for 10 min in room temperature. Cells were washed with cold PBS containing protease inhibitor Cocktail tablets (Roche Molecular Biochemicals, Mannheim, Germany), scraped in PBS, and centrifuged at 2000 *g* for 4 min. Pellets were suspended in SDS lysis buffer. Lysates were incubated 10 min on ice and sonicated to 200- to 1000-base pairs in length on ice. Insoluble material was removed by centrifugation at 13,000 *g* for 10 min, and the supernatant containing chromatin was collected. The supernatant containing chromatin was collected by centrifugation at 13,000 *g* for 10 min. ChIP dilution buffer containing protease inhibitor cocktail tablets was added into each tube containing of chromatin. Samples were preimmunoprecipitated with Protein G Agarose beads for 1 h at 4 °C. After centrifugation at 7000 *g* for 2 min, supernatant was transferred into fresh microfuge tubes. Each sample was immunoprecipitated with 5 μg of specific Nrf2 antibody or normal mouse IgG overnight at 4 °C rotation. Immune complexes were precipitated with Protein G Agarose beads overnight at 4 °C with rotation. Pellet Protein G Agarose beads were washed once with low salt immune complex wash buffer (0.1% SDS, 1% Triton X-100, 2 mM EDTA, 20 mM Tris-HCl (pH 8.1), 150 mM NaCl), once with high salt immune complex wash buffer (0.1% SDS, 1% Triton X-100, 2 mM EDTA, 20 mM Tris-HCl (pH 8.1), 500 mM NaCl), once with LiCl Immune Complex Wash buffer (0.25 M LiCl, 0.5% NP40, 1% deoxycholic acid, 1 mM EDTA, 10 mM Tris-HCl (pH 8.1)), and twice with TE buffer (10 mM Tris-HCl (pH 8.1), 1 mM EDTA). DNA-protein complexes were eluted from Protein G Agarose beads with an elution buffer (0.1 M NaHCO_3_, 1% SDS). Cross-linking was reversed at 65 °C overnight and DNA was extracted using the AccuPrep Genomic DNA Extraction Kit (Bioneer, Daejeon, Korea) according to the manufacturer’s protocol. PCR was performed against the distal E2 (−9.0 kb region) ARE of the *HO-1* promoter (primers 5′-CCCTGCTGAGTAATCCTTTCCCGA-3′ and 5′-ATGTCCCGACTCCAGACTCCA-3′), in reaction buffer. Initial denaturation (5 min at 95 °C) was followed by 40 cycles for 1 min at 94 °C, 1 min at 60 °C, and 1 min at 72 °C. PCR was also performed against the E1 (−4.0 kb region) ARE (primers 5′-CTGCCCAAACCACTTCTGTT-3′ and 5′-ATAAGAAGGCCTCGGTGGAT-3′) and the non-specific region of the *HO-1* promoter (primers 5′-GCTATGTGGGAGGTTGAGGA-3′ and 5′-CCATGGTCAGCAGTTTGCTA-3′). Initial denaturation (5 min at 95 °C) was followed by 40 cycles for 1 min at 94 °C, 1 min at 50 °C, and 1 min at 72 °C.

### 4.10. Generation of Stable Cells Expressing Keap1 Constructs

pBabe parental vector (pBabe-puro-HA-VHL) was purchased from Addgene and Keap1-C151S, -C273S, and -C288S cDNAs were obtained using Muta-DirectTM (iNtRON Biotechnology). PCR-amplified WT Keap1, Keap1-C151S, -C273S, and -C288S cDNA were subcloned into the parental vectors as described previously [60]. To make sure the transfection efficiency of all HA-tagged plasmids in MCF-10A cells, we tried to detect Keap1 protein using the anti-HA antibody (Cell Signaling Technology, Beverly, MA, USA).

### 4.11. BPM-Labeling Assay

DHA-treated cells were washed with PBS and then lysed with RIPA buffer (50 mM Tris-HCl, pH 8; 0.1% SDS; 150 mM NaCl; 1% NP-40; and 0.5% deoxycholic acid). The cell lysate was then centrifuged, and the supernatant incubated with 50 μM BPM for 30 min at 37 °C. The resulting lysates were immunoprecipitated by using avidin-agarose overnight at 4 °C. After being washed, the precipitated proteins were eluted and subjected to Western blot analysis with an anti-Keap1 antibody.

### 4.12. Measurement of Intracellular Accumulation of ROS

To measure the net intracellular accumulation of ROS, a fluorescent probe 2′,7′-DCF-DA was used. Following a 2-h treatment with DHA, cells were washed twice with HBSS solution and loaded with 10 μmol/L of DCF-DA in a 5% CO_2_ incubator kept at 37 °C. After 30-min incubation, cells were washed twice with HBSS solution, suspended in the complete media and examined under a microscope.

### 4.13. Statistical Analysis

Values were expressed as the mean ± SD of the results obtained from at least three independent experiments, and statistical analysis for single comparison was performed using the *t*-test. The criterion for statistical significance was * *p* < 0.05, ** *p* < 0.01 or *** *p* < 0.001.

## 5. Conclusions

In conclusion, our study demonstrates that DHA upregulates the expression of HO-1 and NQO1 in human breast epithelial cells through activation of Nrf2. This is likely to be achieved by activating ROS-mediated PKCδ-Nrf2 signaling and directly modifying critical cysteine thiols present in Keap1 acting as a sensor of ROS and electrophiles.
